# T230. DOSE TRENDS OF ARIPIPRAZOLE FROM 2004 TO 2014 IN PSYCHIATRIC INPATIENTS IN KOREA

**DOI:** 10.1093/schbul/sby016.506

**Published:** 2018-04-01

**Authors:** Young-Joon Kwon, Won-Myong Bahk, Bo-Hyun Yoon, Duk-In Jon, MoonDoo Kim, Beomwoo Nam, Eunsung Lim, Sung-Yong Park

**Affiliations:** 1 Soonchunhyang University Chunan Hospital; 2 Yeouido St. Mary’s Hospital, The Catholic University of Korea; 3 Naju National Hospital; 4 College of Medicine, Hallym University; 5 School of Medicine, Jeju National University; 6 School of Medicine, Konkuk University; 7 Shinsegae Hospital; 8 Keyo Hospital

## Abstract

**Background:**

The purpose of the present study was to evaluate the initial and maximum doses of aripiprazole over a decade to estimate appropriate dosage in clinical practice. We hypothesized that there was a measurable change in dosing patterns during 2004–2014 in Korean psychiatric inpatients.

**Methods:**

In this retrospective study, we reviewed the medical records of patients who were hospitalized in the psychiatric ward of five university hospitals in Korea from March 2004 to December 2014. The patients were at least 18 years of age, prescribed aripiprazole during the index hospitalization and were given at least one prescription for oral aripiprazole. We compared baseline demographic variables among Waves 1 (2004–2006), 2 (2007–2010) and 3 (2011–2014) using univariate one-way analysis of variance (ANOVA) with Bonferroni correction for continuous variables and a chi-square test for categorical variables.

**Results:**

There was a significant difference in mean age among waves (p = 0.012). The use of concomitant medications with aripiprazole was significantly different among waves, as well. The use of other atypical antipsychotics in Wave 1 was 27.0% (n = 20) and 27.4% (n = 55) in Wave 2 and increased to 36.5% (n = 129) in Wave 3, but the difference between Waves 1 and 3 (p = 0.118) and 2 and 3 (p = 0.027) did not reach statistical significance after Bonferroni’s correction. In total, the initial dose of aripiprazole was significantly lower in Wave 3 (7.0 ± 3.9 mg/day) when compared to Waves 1 (10.9 ± 4.6 mg/day, p<0.001) and 2 (10.7 ± 5.6 mg/day, p<0.001). The initial doses of aripiprazole in all diagnostic groups were significantly lower in Wave 3 than in Wave 2.

**Discussion:**

The results from the present study show that the initial doses of aripiprazole, and not the maximum doses, decreased in hospitalized psychiatric patients with the accumulation of clinical experience in aripiprazole use.

